# Rapid diagnostic pathways for suspected colorectal cancer: views of primary and secondary care clinicians on challenges and their potential solutions

**DOI:** 10.1136/bmjopen-2015-008577

**Published:** 2015-10-22

**Authors:** Maria Theresa Redaniel, Matthew Ridd, Richard M Martin, Fareeda Coxon, Mona Jeffreys, Julia Wade

**Affiliations:** 1 NIHR CLAHRC West, University of Bristol, Bristol, UK; 2 School of Social and Community Medicine, University of Bristol, Bristol, UK; 3 Northern Centre for Cancer Care, The Newcastle upon Tyne Hospitals NHS Foundation Trust, Freeman Hospital, Newcastle upon Tyne, UK

## Abstract

**Objectives:**

To ascertain the challenges associated with implementation of the 2-week wait referral criteria and waiting time targets for colorectal cancer and to identify recommendations for improvements to the pathway.

**Design:**

Qualitative research using semistructured interviews and applying thematic analysis using the method of constant comparison.

**Setting:**

10 primary care surgeries and 6 secondary care centres from 3 geographical areas in the England.

**Participants:**

Purposive sample of 24 clinicians (10 general practitioners (GPs), 7 oncologists and 7 colorectal surgeons).

**Results:**

GPs and specialists highlighted delays in patient help-seeking, difficulties applying the colorectal cancer referral criteria due to their low predictive value, and concerns about the stringent application of targets because of potential impact on individual care and associated penalties for breaching. Promoting patient awareness and early presentation, clarifying predictive symptoms, allowing flexibility, optimising resources and maximising care coordination were suggested as improvements.

**Conclusions:**

Challenges during diagnosis and treatment persist, with guidelines and waiting time targets producing the perception of unintended harms at individual and organisational levels. This has led to variations in how guidelines are implemented. These require urgent evaluation, so that effective practices can be adopted more widely.

**Strengths and limitations of this study**

- To our knowledge, this is the first study to explore clinicians’ perceptions of the colorectal cancer pathway, offering a unique insight into its clinical, operational and administrative challenges. These could be invaluable in evaluating, revising and adapting current policies and practices in the clinical setting.
- Our study is broad in geographical scope, encompassing three different regions of the UK, and contrasting the pressing challenges within primary care (making referral decisions in line with the criteria) and secondary care (clinical autonomy).
- We have not interviewed patients and other health professionals, and these groups may have other insights to offer.

## Background

**Figure 1:**
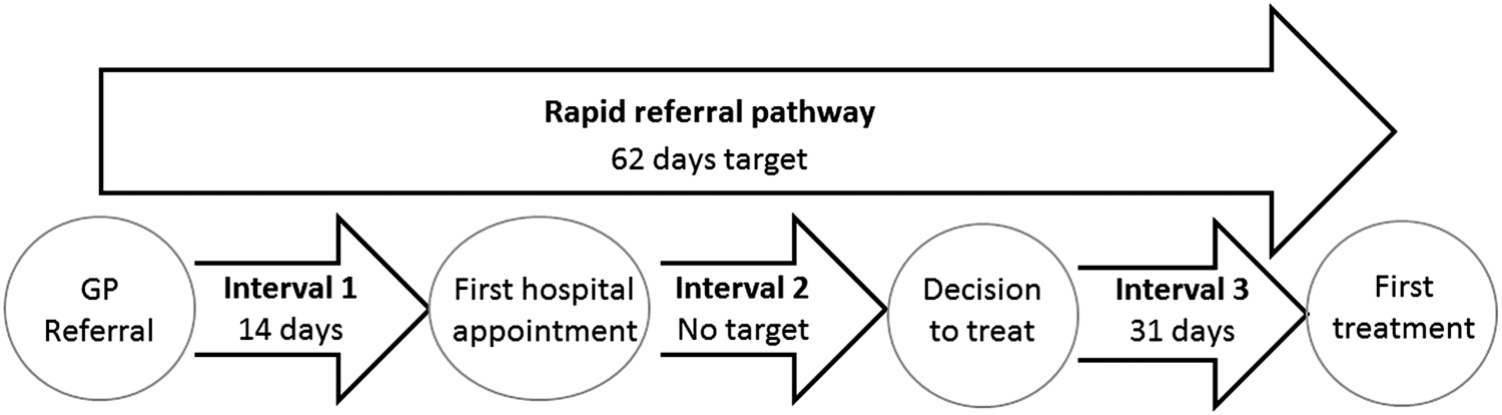
The pathway of patient with cancer (GP, general practitioner).

To address reports that relative cancer survival rates are lower in the UK compared with other European countries, rapid diagnostic and treatment pathways were proposed as part of the 2000 National Health Service (NHS) Cancer Plan.^1^ Targets were set at a maximum 14-day wait between urgent general practitioner (GP) referral for suspected cancer and outpatient appointment in secondary care (the so-called ‘two-week wait’, TWW), a maximum 31-day wait between decision to treat and initiation of treatment and a maximum 62-day wait from urgent GP referral to treatment initiation (figure 1).^1^ ^2^ In parallel, the National Institute for Health and Care Excellence (NICE) specified TWW pathway referral criteria to triage patients with symptoms suggestive of cancer.^3^

Since the launch of the NHS Cancer Plan, audits of the TWW have revealed that rates of referral (ie, the number of GP referrals to the TWW pathway relative to practice size and population served), conversion (the proportion of TWW referrals which result in a cancer diagnosis) and detection (the proportion of TWW referrals among all cancers diagnosed in a GP practice catchment area) vary by primary care practice, suggesting differences in the interpretation and implementation of the referral criteria.^4^ ^5^ In 2013–2014, only 9% of all cancer referrals to the TWW pathway resulted in a diagnosis and treatment of cancer, reflecting the great number but low yield of these TWW referrals.^6^ This is of particular concern for colorectal cancer (CRC), as half of patients diagnosed with CRC do not meet the criteria for referral via the TWW pathway (box 1),^7^ ^8^ and the key alarm symptoms (anaemia, rectal bleeding and abdominal pain) are usually explained by more common conditions, and hence have a low predictive value.^7^

Box 1 **The National Institute for Health and Care Excellence (NICE)-specific recommendations for urgent referral for suspected lower gastrointestinal cancer^3^**

1. Reporting rectal bleeding with a change of bowel habit towards looser stools and/or increased stool frequency persisting for 6 weeks or more, in patients aged 40 years and older.
2. Rectal bleeding persisting for 6 weeks or more without a change in bowel habit and without anal symptoms, in patients aged 60 years and older
3. A change in bowel habit to looser stools and/or more frequent stools persisting for 6 weeks or more without rectal bleeding, in patients 60 years and older
4. Patients presenting with a right lower abdominal mass consistent with involvement of large bowel, irrespective of age
5. Patients presenting with a palpable rectal mass (intraluminal and not pelvic), irrespective of age
6. Unexplained iron deficiency anaemia and a haemoglobin of 11 g/100 mL or below, in men of any age
7. Unexplained iron deficiency anaemia and a haemoglobin of 10 g/100 mL or below, in non-menstruating women of any age.

Criticisms of the waiting time targets have included questioning the strict performance management of secondary care providers and the appropriateness of blanket targets applied to all cancer sites.^6^ At the time of writing, current operational standards for the TWW pathway across all cancers were 93% achievement for the 14-day wait (GP referral for suspicion of cancer to hospital appointment), 96% for the 31-day wait (decision to treat to treatment initiation) and 85% for the 62-day wait (GP referral via the TWW to treatment initiation).^9^ In England (Q3 2014/2015), the accomplishment of the targets for lower gastrointestinal (GI) cancers were 94% and 99% for the 14-day and 31-day targets, respectively.^10^ The 62-day target was met by only 74% of trusts,^10^ with the performance declining from 79% for the same period in the previous year.^11^ While it was acknowledged that this target would only be met 80% of the time for sites with complex pathways such as CRC,^12^ pressure to meet the 85% 62-day target remains and penalties for breaching targets continue to be enforced.

Failure to meet targets for CRC^6^ indicates a need to understand how or why this is occurring. Compliance with guidelines for referral and the achievement of the targets for CRC have been previously assessed in several quantitative studies.^13–15^ One mixed-methods study showed that patients with CRC perceived most delays in the pathway to occur in secondary care.^16^ A mixed-methods study focusing on lung cancer drew attention to clinicians’ and other health professionals’ concerns over secondary and tertiary care capacity issues, inefficient information flows between and within healthcare centres and the lack of leadership within the clinical team.^17^ In a qualitative study focusing on breast cancer, GPs and specialists acknowledged the difficulties of making the correct decision on referral based on presenting symptoms alone.^18^

To our knowledge, no previous study has involved an in-depth analysis of relevant clinicians’ perspectives on the entire CRC pathway from referral to start of treatment. There is a need to assess problems arising across geographic areas and different clinical specialties (primary care, surgery and oncology) and possible solutions to the challenges. We interviewed primary and secondary care clinicians to ascertain their views about patient pathways in CRC, the challenges associated with implementation of the TWW referral criteria and waiting time targets for CRC, and their recommendations for improvements to the pathway.

## Methods

### Recruitment and sampling

In-depth interviews with GPs, oncologists and surgeons were conducted in three cities, each from different regions in England (north, midlands and south). Clinicians were purposively sampled to include those in areas with the longest and shortest waiting times and corresponding high or low excess mortality,^19^ and to represent a range of primary and secondary care experience (maximum variation sampling). The final sample size was determined by data saturation, that is, when no new themes emerged from three successive interviews.

Primary care trusts (PCT) initially, and later clinical commissioning groups (CCGs), facilitated sampling among the GPs. Eligible GPs were first identified and contacted by PCTs or CCGs. Those GPs who expressed an interest in participating were then contacted by email. Surgeons and oncologists were approached directly via email in two of the three sites, their contact details having been obtained from their NHS Trust's websites or from the oncology and lower GI surgery administrators. In the third study area, CRC surgeons and oncologists were recruited through the Trust Research and Development Unit, with those who had expressed interest being contacted via email. All clinicians received a letter of invitation and participant information sheet attached to the email. Of those contacted, 22 did not reply to the introductory email (4 GPs, 1 oncologist, 17 surgeons) and 4 refused to participate due to clinical load (3 GPs, 1 oncologist).

### Data collection

All interviews were conducted by MTR between March 2013 and February 2014. Twenty-one were face-to-face interviews in GP practices or in hospitals and three were telephone interviews. Topic guides ensured that issues identified by our research group were raised, if time permitted, while also allowing participants to raise relevant issues not included in the topic guide. Key issues covered in the topic guide included: (1) views on the cancer referral-to-treatment pathways; (2) factors perceived to affect the CRC waiting times (patient, clinician-related and system-related); (3) views on the impact of waiting times from referral to treatment on cancer outcomes; and (4) clinicians’ views on possible interventions to improve adherence to waiting time targets.

Interviews were audio-recorded and transcribed verbatim, with the exception of one interview where the audio-recording was of poor quality. Extensive notes made during and after the interview were used instead. All audio-recordings and transcripts were anonymised. Audio-recordings were transcribed into the NVIVO software.

### Data analysis

Analysis was carried out concurrently with data collection and informed sampling and further data collection (interviews). Interviews were transcribed and coded shortly after data collection. Codes were assigned by MTR to phrases that captured each concept conveyed by the clinician, and codes were grouped under common headings or themes. A sample of the transcripts (N=9) were independently coded by two researchers (MTR and JW), and coding and thematic headings were modified following discussion of differences in interpretation. A third researcher (MJ) also read a sample of the transcripts (N=7) and contributed to the development of the thematic headings. Further sampling was undertaken to explore the emerging concepts and themes. We conducted a thematic analysis using the method of constant comparison^20^ where codes were refined inductively and iteratively based on discussions by the research team in response to new information emerging from subsequent interviews. Transcripts were encoded using NVIVO V.10 software^21^ to facilitate coding, retrieval and analysis of data.

**Figure 2:**
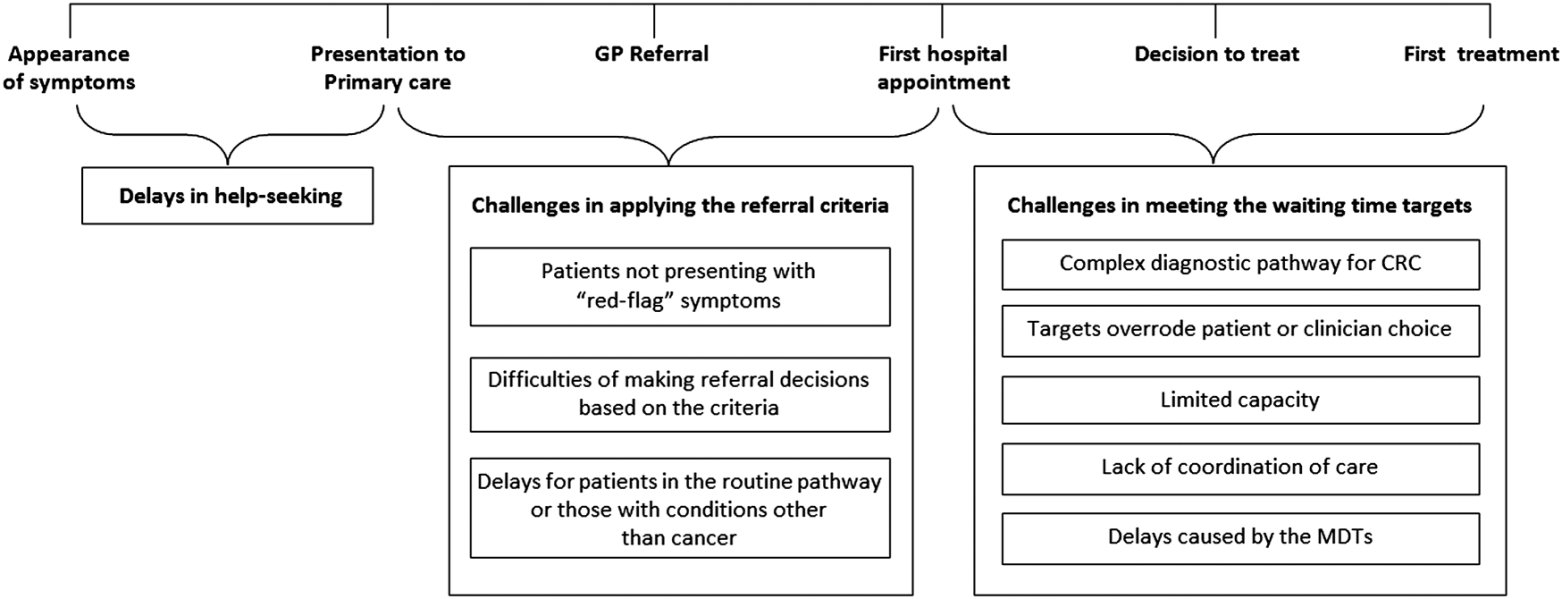
Challenges associated with implementing the referral system and meeting operational standards. CRC, colorectal cancer; GP, general practitioner; MDT, multidisciplinary team.

Data across participants were systematically compared to identify similarities and differences in clinician perspectives according to a range of clinician characteristics (eg, clinical specialty, geographical area and years in post). Matrices with summaries of participant responses across emerging themes were created to aid identification of similarities and contrasts according to these characteristics. A diagram illustrating how these themes related to time points or intervals in the pathway (appearance of symptoms, presentation to primary care, GP referral, first hospital appointment, decision to treat, first treatment) was created (figure 2).

## Results

We interviewed 24 clinicians (10 GPs, 7 oncologists and 7 surgeons) from the three different areas. Ten clinicians were interviewed in area 1 and seven each for areas 2 and 3 (the areas are not named to preserve the anonymity of the people we interviewed). A summary of the clinicians’ profiles is shown in table 1.

**Table 1:** Sociodemographic characteristics of clinicians in the study

Variable	N	Area 1	Area 2	Area 3
Specialism, N				
Primary care	10	4	3	3
Oncology	7	3	3	1
Surgery	7	3	1	3
Gender, N				
Male	15	4	6	5
Female	9	6	1	2
Years in post, mean (SD)				
Primary care	10	11.4 (8.8)	9.7 (11.7)	8.5 (9.5)
Oncology	7	12.2 (5.8)	11.7 (5.0)	14.0 (0.0)
Surgery	7	9.3 (2.1)	17.0 (0.0)	12.0 (9.2)
Years in specialty, mean (SD)				
Primary care	10	18.8 (5.0)	15.3 (6.8)	12.2 (6.5)
Oncology	7	20.7 (9.8)	10.5 (0.7)	14.0 (0.0)
Surgery	7	11.7 (5.7)	17.0 (0.0)	12.5 (8.4)
GP surgery SES profile,* N				
Low deprivation (IMD 8–10)	1	0	0	1
Medium (IMD 4–7)	4	2	1	1
High deprivation (IMD 1–3)	5	2	2	1

*For 10 GPs interviewed, based on the IMD profile of the area.^22^

GP, general practitioner; IMD, Index of Multiple Deprivation.

Clinicians were asked to describe the typical stages of the pathway from GP referral to treatment and their perception of the influence of referral guidelines and waiting time targets. As interviews were conducted, it became apparent that clinicians spontaneously and repeatedly highlighted the importance of the period prior to GP referral: the time taken to present to the GP with symptoms and difficulties for clinicians in interpreting the referral criteria. In the interest of presenting a holistic analysis, these issues have been included in the findings.

The results are presented in three sections: positive impacts of the TWW referral and waiting time targets, challenges associated with implementing the referral system and meeting operational standards, and strategies to improve systems for rapid diagnosis and treatment for CRC.

### ‘Overall it's been very valuable’: positive impacts of TWW referral and waiting time targets

Clinicians from the all areas and all specialties had generally favourable opinions on the TWW targets and guidelines, believing they reduced waiting times for tests, diagnoses and treatment, and reduced anxiety for patients.

I think overall it’s been very valuable and it is reducing the numbers of people who are dying with advanced colon cancer and I think it is making the whole journey that you have with colon cancer in that you don’t have a long stressful time, waiting to be seen, waiting for your results, waiting for your operation which used to drive a lot of people to pay loads of money to go privately. Surgeon 1 (Area 1)

They also provided security for clinicians and served as an explicit public standard for clinical practice:

I think it’s also nice knowing that if you are at all worried they’ll definitely be seen within those 2 weeks. I think it’s also reassuring to know that treatment will be instigated within a certain period of time. GP1 (Area 1)

It does give you some security…when they [patient] say why can’t I have my operation next week and you say well we need to treat you, the guidelines are we need to treat you between, before this date and that’s the same everywhere and as long as we do that that’s considered reasonable. Surgeon 5 (Area 3)

### Challenges associated with implementing the referral system and meeting operational standards

Clinicians raised various challenges associated with adhering to waiting time targets (figure 2). They also drew attention to delays arising prior to patient presentation and difficulties interpreting and applying the rapid referral criteria for CRC.

#### Delays to help-seeking: *‘It's about the period of time the patients been at home with their symptoms’*

All clinicians, particularly GPs, mentioned that the patient journey prior to GP referral was not covered by the cancer waiting time guidelines, yet might potentially have significant impact on outcome. This included patient delay in seeking GP advice after symptoms appeared and the wait for a GP appointment. Clinicians argued that time between appearance of symptoms and patient presentation to the GP accounted for most of the delay in the pathway of patient with cancer, negating efforts to expedite the diagnosis from the point of referral:

I think that what the problem is more likely to be is how long it takes people to present to us and how long it then takes us to think they need that sort of investigation. GP1 (Area 1)

I know from trying to see my own GP that actually it can be 3 or 4 weeks to see your GP of choice and then you’re into the 2 week wait system. GP4 (Area 1)

It’s not uncommon that you’ve got a patient who’s been at home with their symptoms for months so it does kind of make a slight mockery of things then that you have to get on and actually investigate them and treat them within their set period of time because actually that’s, the issue isn’t about that period of time it’s about the period of time the patient’s been at home with their symptoms. Oncologist 5 (Area 2)

#### Challenges applying the referral criteria for TWW referral

##### ‘Patients don't always present with those red flags’

All groups highlighted the problems applying the TWW referral criteria for CRC (box 1) to individual patients. GPs reported difficulty applying the referral guidelines, particularly when patients presented with non-specific symptoms, and for those with comorbidities:

[diagnosis is difficult for] people who may come with very vague symptoms. People who may have had a diagnosis of a previous bowel problems…Patients with other multimobidity, comobidities so it’s difficult to decide whether it’s related to a potential new diagnosis or it’s already related to some other factor of that…sometimes patients don’t always present with those red flags…you have difficulty getting that patient seen within 2 weeks because they don’t fit the criteria. GP7 (Area 2)

Specialists acknowledged the difficulty of applying the guidelines and noted that GPs encounter large numbers of patients with non-specific but potentially cancerous symptoms, out of whom only a very small proportion will eventually be diagnosed with cancer:

For colorectal cancer GI symptoms are very common within the population…and if you think about the GP they may see less than 1 patient a year with bowel cancer so for them it’s hard to pick out the cancer from the non-cancer. Surgeon 3 (Area 1)

Some surgeons and oncologists questioned the evidence base underlying use of duration of presenting symptoms as a predictor of outcome. Symptoms might become suddenly apparent in rapidly advancing late stage disease, which in turn was likely to have a poor outcome irrespective of time to diagnosis or treatment:

The trouble with the 2 week wait is that the premise is fundamentally flawed, because the assumption is that [it's] the length of symptoms of colorectal disease, the stage of disease at presentation which is what determines outcomes and there’s no evidence to support that. Surgeon 4 (Area 2)

Paradoxically the people with a long history of symptoms often do better than those with a short history of symptoms and that’s because you can have a slow growing indolent cancer that gives you a bit of symptoms for a long time but it’s actually not that aggressive and sometimes you can have quite an aggressive cancer that gives you symptoms very quickly. Surgeon 3 (Area 1)

##### *To refer or not to refer:* fears of ‘clogging up the system’ versus ‘being negligent’

In all three geographical areas, there was reported variation in referral practices. Some GPs were happy to refer patients under the TWW pathway about whom they had concerns, but who did not strictly meet the TWW criteria, sometimes with the complicit agreement of secondary care specialists. Others felt unable to do so despite having serious concerns that a patient might have a cancer:

The dilemma is if you have somebody who you think it’s not quite right but I can’t quite tick all the boxes on my 2 wait week form and I don’t want to use a 2 week wait system if it’s not appropriate because that means somebody else with cancer potentially might have to wait longer. GP2 (Area 1)

I know the consultants well enough to be able to phone them up and say it’s out of the the 2 week rule guidelines but they really need to be seen and I’ve done that several times and the consultants have seen them. Or they’ve said just stick them on the 2 week rule pathway… GP8 (Area 3)

GPs also raised the issue of having to refer patients who fulfilled the criteria but for whom the probability of cancer was low: non-referral was deemed negligent, but the practice undermined professional judgement and was a poor use of resources.

I possibly may end up referring patients who I don’t necessarily feel clinically might have, say bowel cancer, but they do fulfil the list so there’s a possibility that in my clinical experience I would say that I thought it unlikely but they fulfil the list so to not refer them I would perceive as being negligent. GP5 (Area 2)

If somebody comes in with something…even if I really don’t think it’s cancer if they are on the 2 week rule pathway it’s great but because there’s no other action for me to take. Because if I don’t refer them that’s really quite negligent even if I don’t think it is something. GP8 (Area 3)

The number of patients we have to see to rule the cancer out has increased, so you actually end up seeing a lot more non- cancer patients to get a very small percentage…the system is going to get clogged, that will invariably delay things for genuine 2 week wait. Oncologist 6 (Area 2)

Some surgeons felt that certain GPs abused the system, did not adhere to the TWW criteria and at times referred too quickly. Another surgeon expressed a preference for increased referrals as opposed to running the risk of missing a cancer diagnosis:

They [GPs] are seeing a pathway that they can manipulate to get their patients seen as quickly as possible, knowing that the chance of their patient having bowel cancer is very very low or probably negligible but they are working for their patients so were clogging up the system. Surgeon 1 (Area 1)

Patients should have symptoms for 6 weeks or more before referral and actually primary care does not adhere to that…the minute they [GPs] see someone with rectal bleeding they might be very nervous and send them up and they ignore the pathway because they think it could be cancer. Surgeon 3 (Area 1)

I would much rather I was over-referred rather than a filter was put in and we were under referred…I’d rather not miss a patient…I don’t care if they [GPs] get it wrong. It’s getting the people. Surgeon 6 (Area 3)

##### *Unintended consequences:* ‘we’re disadvantaging all the other patients’

GPs and specialists argued that the implementation of the referral criteria resulted in an increased burden on the hospital list for tests, with the unintended consequence of an increase in the waiting time of patients on the routine pathway, or those with conditions other than cancer:

People who didn’t fit that criteria or didn’t get sent by that criteria were very much disadvantaged by it because they were then being delayed because we had to make all this capacity which we didn’t have to hit the 2 week wait which then meant anybody else were in the other pool and they were delayed by months. Surgeon 2 (Area 1)

We are seeing vast numbers of people only a small percentage turn out to have cancer and we’re disadvantaging all the other patients like people with inflammatory bowel disease and the multitude of other patients that need care and need scanning and so on that they are delayed now. Oncologist 4 (Area 2)

An oncologist expressed concern that the referral criteria might be increasing the threshold of GPs against referring patients that fall outside of the criteria:

The trouble is there always will be people who are out of the box and having those 2 week wait forms makes it seem that people with other symptoms are less likely to have malignancy and maybe reduces the threshold for referring them so. Oncologist 1 (Area 1)

#### Challenges meeting waiting time targets for diagnosis and treatment

##### ‘CRC is the worst of all our targets because it is more complicated’

Oncologists argued that the waiting time targets did not take into consideration complexities associated with diagnostic tests for CRC, such as the need for bowel preparation prior to endoscopy, the procedure itself, the number of tests needed and the length of the waiting time for each. A common criticism among clinicians from all specialties was that the waiting time targets take a ‘one-size fits all’ approach, when in fact cancer is a complex and diverse disease, and a standard that was devised for one cancer site (eg, breast cancer) might not be applicable to CRC:

Colorectal is the worst of all our targets because it is more complicated, you’ve got to do an endoscopy and often a scan…the added complexity with bowel is you’re having to do an invasive investigation in an endoscopy and that is time consuming. It relies on the patient getting proper bowel preparation. Oncologist 4 (Area 2)

##### *Waiting time targets over-rode patient or clinician choice:* ‘the patient is like something in a factory’

Once the patients were referred via the TWW pathway, both the GPs and specialists felt that meeting the targets sometimes over-rode patient or clinician choice. They noted that patients did not have any input in the timing of tests and treatments, which were driven by the targets. Clinicians argued that problems with patient choice mostly applied to those who wanted to delay the process for reasons such as competing priorities in a person's life, or those presenting with complex disease (usually patients who were older, difficult to diagnose, manage or treat, or involving multiple specialties):

I don’t know that the patient has any control whatsoever over what happens…it’s just a juggernaut that they have no input in timings. They have input in what’s going to happen but they don’t have any input in timings. GP4 (Area 1)

It is just here is a patient, it moves through a conveyor belt. The patient is like something in a factory, an object being managed, processed, manufactured on a conveyor belt through a factory and once you are on it nothing, it seems like nothing is allowed to alter the process…Patient choice is not allowed and it makes it feel very rigid and very difficult for a few patients, not many. Surgeon 1 (Area 1)

Specialists perceived themselves to be under considerable pressure to meet targets because hospital NHS Trusts were subject to financial penalties for target breaches. Specialists also saw the emphasis on meeting the standards as detrimental to setting targets for individual patients, resulting in potentially compromised care:

We’ve got people on our back all the time telling you you’ve get this patient in, you’ve got to operate before this date, they’ve got to have the chemotherapy or radiotherapy before that date. Surgeon 5 (Area 3)

You can’t say look this patient who’s out there nearing their wait time but doesn’t actually need to start tomorrow can be delayed a week and reach their wait time whereas this patient who’s got another 2 or 3 weeks to their wait time deadline needs to start tomorrow, put them in their place and treat them or delay. You can’t, you don’t have that choice. Oncologist 3 (Area 1)

I’d like them [patients] to go into a clinical trial but if they go into the clinical trial they’ll definitely breach. But I think the clinical trial is in the best interests of the patient so what do I do? If I put them in the trial they’ll breach and we’ll get a financial penalty. If I don’t put them in the trial I think they’ll potentially getting substandard care but they won’t breach. Oncologist 5 (Area 2)

A GP argued specialists should be allowed to set flexible targets when necessary:

I think that consultants should be allowed to stipulate whether something can be dealt with outside those standards…if they see somebody quickly, make the diagnosis but then decide that actually that person’s treatment doesn’t need to start in preference to somebody else because they feel that that’s absolutely safe and it’s not going to benefit them to be starting treatment sooner…I think they should be allowed to say that. GP1 (Area 1)

Surgeons reported over-riding the guidelines when they felt it necessary to do so in the patient's interest:

There are occasional patients who say ‘I want to go away and think about things’ or ‘I want to go away and have a second opinion’ or ‘I want to go away and discuss with family or friends’…some patients do ask for a bit more time than the pathway recommends and I would never go against the patients’ wishes on that. Surgeon 1 (Area 1)

I’ll take them [patients with complex pathways] out of the pathway and I don’t care what anyone says to me. The care of the patient and the survival of the patient at any point of their contact with me is more important than any administrator's pathways. Surgeon 6 (Area 3)

##### *Capacity:* ‘we don't have any slack in the system’

For most clinicians in the three geographical areas and from the three specialties, a key challenge was limited capacity in secondary care (potentially compounded by increased referrals described above). This encompassed consultant clinics, slots for tests and scans, and theatre lists.

One of the main obstacles in the area where I work is the long waiting list…I think some in gastroenterology have waitlists as long as 3 months. GP10 (Area 3)

We don’t have any slack in the system so if we had a sudden bulge in the 2 week wait referrals we have very little room to increase our capacity. Surgeon 1 (Area 1)

I sometimes just have to over book patients so that they don’t breach. Oncologist 2 (Area 1).

##### *Coordination of care:* ‘this poor patient is shunted from person to person’

A lack of coordination within the healthcare system was highlighted by GPs from all three geographical areas and one oncologist (area 1). Communication problems between primary and secondary care, the different departments in secondary care, and between private healthcare providers and the NHS led to delays in transmitting test results and other relevant patient information. GPs suggested that increased specialisation had led to compartmentalisation of care, and to patients with complex problems being endlessly referred from one specialism to another without resolution, if the problems do not fit a particular field:

In the old days when we referred when there were no pathways there was often a relationship between the GPs and the specialist so I would know the surgeon and I would ring up and explain the problem… and nowadays it's much harder. GP4 (Area 1)

2 week waits can be great initially but I think they can actually lead to delays because of [secondary care] people saying well that’s not my department, that’s not my department, and this poor patient is shunted from person to person. GP1 (Area 1)

I say I’m quite lucky because the vast majority of consultants I can ring them up…and I think because we’re quite a small area, the GPs tend to know most of the hospital consultants and the hospital consultants know most of the GPs…I think if I worked in an area where I didn’t know the consultants or if they didn’t know me so if I was a locum GP or a new GP to the area or didn’t know which consultants I kind of rely on then, that might be completely different. GP8 (Area 3)

GPs and specialists reported that while patients were informed of the referral, diagnosis and treatment process, which clinician had responsibility for an individual patient's care became unclear once patients were in secondary care, for example, where there is onward referral and the patient is seen by different doctors for different parts of the referral process. All clinicians agreed that there were times when the patient did not meet the person responsible for their overall care until later in the pathway:

They [patients] might be seen by one doctor originally and then be told their diagnosis by another and then surgery discussed by another just to try to get them through the system quickly. Surgeon 2 (Area 1)

It’s multiple levels of referral so until you actually get the final diagnosis, the final scan you don’t actually see that person who’s going to be responsible for your care. Oncologist 6 (Area 2).

##### Potential for death by MDT

Both GPs and specialists raised issues pertaining to multidisciplinary teams (MDT), where shared decision-making on the patient's management took place. The MDTs were mostly seen as a positive, but had the potential to cause delays in some cases:

I can understand why it’s really good to have lots of opinions to get the right treatment for patient…one of the problems that an MDT brings, it brings lack of ownership…it seems that at that point it’s very difficult to know who’s in charge and what’s happening and it takes some time to get the results out of what’s happened from the MDT meeting and that seems to be a bit of a stalling point. GP4 (Area 1)

MDTs are meant to be good but there’s no doubt there’s potential for death by MDT…you are just going from 1 person to another so if you’ve got a possible something in the liver it goes to the liver MDT or the communication has to come back or a lung MDT and back again so there’s all this toing and froing and often the patient gets lost in the middle… Surgeon 2 (Area 1)

In certain cases, it was felt that decisions by an individual clinician should suffice and the wait for the MDT meeting was deemed unnecessary:

The other thing is that some physicians will say, they won’t make a decision, they’ll wait for the MDT which is weekly and it might be you’ve just missed the MDT so you’ve got 6 days, whereas in fact somebody could have made a decision; it didn’t really need to wait for the MDT. Oncologist 5 (Area 2).

### Strategies to improve systems for rapid diagnosis and treatment for CRC: recommendations and examples of existing practice

Clinicians made several suggestions to improve current systems for rapid diagnosis and treatment of CRC, and gave examples of measures implemented in their areas (table 2). These were classified into four themes: promoting awareness and early presentation; review of the criteria for rapid referral and how they are implemented; optimising limited resources; and facilitating continuity of care.

**Table 2:** Strategies to improve the waiting time system: recommendations and examples

Themes	Challenge(s) to which recommendation apply	Recommended strategies	Examples
Promoting patient awareness and early presentation	Delays in help-seeking	Have a positive message about cancer treatments Patient education Invest in screening and early diagnosis	Adverts for cancer (area 1)
Review of the criteria for rapid referral	Patients not presenting with ‘red flag’ symptoms Difficulties of making referral decisions based on the criteria Delays for patients in the routine pathway or those with conditions other than cancer Complex pathway for CRC diagnosis Targets over-rode patient or clinician choice	Refine the CRC rapid referral criteria Increase GP access to investigations Referral options for patients outside the guidelines Decide on patient timelines based on clinical need or clinical suspicion of cancer Make allowances for patient choice Have a single time scale for all patients Revise the waiting time targets for CRC Change the penalties system for breaches	Feedback to GPs on results of referrals (area 3)
Optimise limited resources	Limited capacity	Increase capacity Have designated diagnostic centres Shift some investigations to primary care Create nurse-led clinics Have flexible or dedicated TWW lists for tests	Assignment of some investigations to primary care (area 2) Increase in the number of colorectal surgeons (area 2) Increase in endoscopy capacity (area 3) Dedicated diagnostic imaging slots (area 2)
Facilitate coordination of care	Lack of coordination of care Delays caused by the MDTs	Named consultant responsible for the patient once referred Automate the system Give patients appointment reminders Use of support services Obtain complete patient information Create one-stop clinics Have straight to test options Expedite the MDT process for some patients	Online referral system (Area 2) Provision of nursing support for patients (area 1) Improvement in triage of complex patients (area 1) Clearer instructions for preparation for tests (area 2)

CRC, colorectal cancer; GP, general practitioner; MDT, multidisciplinary team; TWW, two-week wait.

#### Promote ‘patient awareness’ and early presentation

Clinicians reiterated the importance of encouraging patient help-seeking behaviour and of expediting early presentation to the GP:

[patients] have to overcome the fear and negative ideas that if you have cancer you are going to die, we need a more positive approach. GP10 (Area 3)

I think that there could be more patient alert adverts. And for example when there are bowel adverts or lung adverts we know the referrals increase to the hospital. So if you had more patient awareness then the patients would go to the GPs and be referred in. Oncologist 2 (Area 1)

You could argue that actually you could (put) more money into early diagnosis so the screening programme's doing that. Surgeon 3 (Area 1).

#### Review the criteria for rapid referral and the waiting time targets

##### ‘What are the predictive symptoms? Get that right first’

Applying the criteria for rapid referral was viewed by clinicians from all three groups (GPs, surgeons, oncologists) as a key challenge. GPs and specialists focused on the need to improve the TWW rates of conversion and detection. Suggested ways to do this included: (1) research to improve the positive predictive value of the referral criteria; (2) increasing direct access to investigations by GPs; or (3) by providing GPs with feedback on their individual conversion and detection rates. Examples of the last intervention had been implemented in some areas and were felt to have improved the accuracy of primary care referral:

When they are seen in clinic that’s the problem because they don’t have those symptoms so…firstly to get the, what are the predictive symptoms, get that right first. Oncologist 1 (Area 1)

We could be more targeted than our 2 week wait referrals if we could do some investigations and get them done quickly in the community. GP2 (Area 1)

I get a visit every year, every 6 months from the cancer lead…and he came to see and what he does is he compares your hit rate, what your hit rate is, then he asks how many of your cancers have been diagnosed through emergency admissions. GP8 (Area 3).

##### More flexibility in the system

Specialists recommended greater clinical autonomy in determining both who to refer via the TWW pathway and which waiting time targets were relevant for individual patients:

It would be better if there was that leeway, that kind of box…if your level of suspicion is high or you do suspect cancer. GP3 (Area 1)

What I would rather have is a more flexibility in the system so that we had stratification of the wait time…whereby for the patients with a specific cancer diagnosis where it [waiting time to treatment] was likely to make a difference that actually time scales were squeezed and brought down, and for patients where actually it’s not going to make a difference, that there was more flexibility to actually push them out but probably with a full bag, minimum time that we are comfortable with. Oncologist 3 (Area 1)

Clinicians felt there was also a need to allow patients flexibility in the timing of their journey through the pathway, particularly for those who needed time to come to terms with the diagnosis or to consider the side effects of available treatment options:

Everybody is fixed on the target and the target could not be more important than the patient. GP10 (Area 3)

To try and push people through in weeks when they haven’t had a chance to think about what you’ve told them, you know having a stoma, should you have a stoma, functional problems with surgery, risks to chemotherapy, risks to this, risks to that, I think the process shouldn’t drive that, it should be the other way round. Surgeon 2 (Area 1)

Suggestions from specialists included a single time frame (either 2 or 6 weeks) for all cancer referrals, not just those that meet the TWW criteria or lowering the accomplishment of the 31-day and 62-day targets (currently 96% and 85%, respectively^9^) or changing the penalties system:

Let’s see them [patients] all within 2 weeks, everybody. Surgeon 1 (Area 1)

It might be much better to say actually all patients need to be seen in 6 weeks. Surgeon 3 (Area 1)

I would slacken the demand on the 90% 31 day and 62 day targets. Surgeon 1 (Area 1)

I wouldn’t fine people…you are failing because you can’t meet the guidelines and that’s generally because you haven’t got enough infrastructure and then if they fine you for not meeting it you’ve got even less infrastructure. Oncologist 4 (Area 2)

It would be nice if there was a reward as well as a stick…that if you got the people treated sooner than the 62 days that there was some financial incentive. Oncologist 7 (Area 3).

#### Optimise limited resources

Most clinicians attributed the problems meeting the waiting time targets for the TWW pathway to limitations on capacity, particularly in secondary care. Several suggested increasing the capacity in terms of staff (doctors, nurses and radiologists), slots for tests and operating theatres, and facilities (scanners). Such initiatives were already perceived to have been effective in some areas:

In order for those peaks of demand to be met you would need to have a lot more capacity and there would be times when it wasn’t being used but if you really wanted to meet all of that demand you would have to build in that spare capacity. Oncologist 7 (Area 3)

About a year ago when we were in real trouble because we were so behind so they appointed new colorectal surgeons and new nurses. They put a lot of money into improving it [waiting times]. Oncologist 4 (Area 2)

They have increased the endoscopy treatment so it has responded. We are doing CT colons now which we didn’t used to do and that’s partly because of the waiting time so the services have improved. Surgeon 7 (Area 3)

Other suggestions focused on optimising current capacity, including having designated diagnostic centres either within the NHS or in the private sector, the creation of nurse-led clinics, shifting some investigations into primary care (see above) and having flexible lists and dedicated slots for TWW patients to expedite the process. In some areas, the latter two interventions were already perceived to have expedited the diagnostic process and helped avoid breaches.

Maybe the diagnostic part could be out sourced to a private company so that, where we do the 2 week referral…they can quickly see if there is a cancer or not. GP6 (Area 2)

I think the whole process for the under 65s of people who have no comorbidities or people who have no other major medical problems should be automated through a nurse led clinic… Surgeon 6 (Area 3)

Also one of the things that’s holding up the scans sometimes was that the patients hadn’t got their urea and electrolytes checked…so when they are sent by the GP, the GPs are asked to check their bloods so that result is ready when they arrive. Oncologist 4 (Area 2)

We found that to speed it [waiting time] up we’d have to create some dedicated imaging slots for them [TWW patients]. Oncologist 4 (Area 2).

#### Facilitate coordination of care

Most clinicians felt that improved coordination of care would help meet the waiting time targets. GPs from area 1 recommended having a lead clinician who would be responsible for the patient while in secondary care. They also recommended the establishment of an automated system that would allow online referrals and feedback, and enable GPs to track electronically their patients’ progress through the pathway:

I think that within all of this it has to be very clear who the lead clinician [secondary care] is because if you have generic departments making referrals for something like the 2 week wait you need to be absolutely sure that…a named person is getting the results and is dealing with that because that would be a possibility for error to come in. GP1 (Area 1)

What would be really good is if there was some central database or spreadsheets, or admin type record that stated exactly what was happening…so we could follow the patient's journey. GP4 (Area 1)

I know they moved to try to speed that up by making it electronic…I think that’s successful, because…we are markedly improving our 2 week percentages. Oncologist 4 (Area 2)

Recommendations also included facilitating the flow of patients from referral to treatment with strategies such as giving patients appointment reminders (card or telephone calls), using support services (language interpretation services for non-English speakers, nursing support), obtaining complete patient information at the beginning of the pathway, having one-stop assessment clinics or sending patients straight to diagnostic tests rather than via the specialist.

I think that more information has to be gleaned about the patient at an early stage so they can be directed into the most appropriate use of resource. Oncologist 5 (Area 2)

I would be looking more towards integrated appointments so for example if you go and have a colonoscopy you may have your CT at the same stage and same time your blood’s done so then all the investigations are done at one go. GP4 (Area 1)

I would try and shorten the length of time to investigation which would involve people going straight for tests. GP6 (Area 2)

To improve patient flow through the diagnostic pathway, some patients were given nursing support (area 1) or clearer instructions for preparations for tests (area 2). In area 1, an acute oncology unit helped improve triage of complex patients.

I gather that [bowel preparation instruction] markedly improved the quality of the preparation of the bowel so that they were far more likely to be, have a successful endoscopy…they are sent the preparation and they are told you know at 9am do this do that and if you have a problem phone us and so on and so forth. Oncologist 4 (Area 2)

The patients who used to be more of an issue are the ones who presented with metastatic disease as an emergency…often as a frail perhaps patient or an elderly patient or a patient with other comorbidities…they’d often then get referred to all sorts of different areas within the hospital perhaps simultaneously, and lots of tests done without really thinking and focusing on what would make a difference to the patient’s outcome…now with the acute oncology…they can be seen immediately and advice can be given. Oncologist 3 (Area 1)

A GP from area 1 proposed expediting the MDT process for some patients, particularly those whose management was straightforward. This meant being included in the test, preoperative or surgery lists while waiting for the MDT meeting:

I don’t see why they can’t be listed for surgery and start the work up even if from listing to surgery there’s a MDT meeting in the middle or whether you could just be checked, and that would speed things up for the patient, you know rather than wait for the MDT meeting, then list them, then they need pre-op, and then they need the surgery. GP4 (Area 1).

## Discussion

### Principal findings

The introduction of the rapid diagnostic pathway (TWW) and associated maximum target waiting times was believed by clinicians to have had a generally positive impact on patient care. However, more than 10 years after the introduction of the TWW, GPs and specialists highlighted ongoing challenges associated with its implementation. In addition to delays in patient help-seeking, clinicians highlighted difficulties applying the TWW referral criteria for CRC, and concerns over the stringent application of waiting time targets (and associated penalties for breaching targets) which were perceived as potentially compromising patient-centred care. Promoting patient awareness and early presentation, identifying the predictive symptoms, allowing flexibility, optimising the use of resources and facilitating the patient journey through the healthcare system may improve the implementation of the cancer TWW guidelines.

### Strengths and weaknesses of the study

To our knowledge, this is the first study to explore clinicians’ perceptions of the challenges associated with the 2-week wait pathway for CRC. It is also broad in its geographical scope, encompassing three different regions in the UK. Our sample included clinicians from a range of specialties (GP, colorectal surgery, oncology) and a range of locations and there was considerable agreement across specialties and geographical areas as to what challenges there were and how these might be addressed. Our results differentiated between challenges that are pressing for GPs (making referral decisions in line with the criteria) and for specialists (clinical autonomy). Limitations include not interviewing patients or other health professionals such as nurses, managers and laboratory personnel. These groups may have suggested other interventions to improve adherence to waiting time targets that may have been salient to these groups.

Our results were in agreement with reported clinician views in the context of breast and lung cancers,^6^ ^17^ ^18^ but we also highlighted other concerns such as the role of complex diagnostic pathways for CRC and the potential negative impact of the waiting time targets on patient choice and clinical autonomy. Clinicians in our study identified strategies to reduce the delay in the CRC pathway which have also been cited in various studies.^23^ They offered additional recommendations (eg, having a named consultant assigned for the patient, increasing GP access to investigations, referral options for patients outside the guidelines, giving GPs access to an online system to track patient progress through secondary care) and cited examples of practice implemented in their areas, the effectiveness of which will need further investigation.

### Possible explanations and implications for clinicians and policymakers

Our study highlights the challenges faced by clinicians in implementing the referral guidelines, which may result in inappropriate referral, and low conversion and detection rates. Audits have shown that up to 30% of patients with CRC meeting the TWW referral criteria were regarded as ‘inappropriate referrals’, defined as referrals to the TWW route who were found to have no cancer.^14^ Compliance with the referral guidelines varies and non-compliance has been suggested as a reason for the low detection rates.^5^ ^14^ However, some ‘inappropriate referrals’ included patients who met the referral criteria,^14^ suggesting that poor compliance was partly attributable to the low predictive value of the red flag symptoms.^5^ ^14^ ^24^ This is supported by evidence from quantitative studies that imply that some symptoms are markers of more biologically aggressive tumours that present earlier while some symptoms only appear in later stages of the disease.^25^ ^26^ These further complicate the challenges of applying the referral criteria and some of the assumptions it makes about the biological behaviour of CRCs. Tools to aid cancer symptom appraisal and referral have been proposed and evaluated, with limited success.^23^ Other interventions were suggested by participants in our study, including a review of the referral criteria and increased access to diagnostic tests by GPs; the applicability of these in practice should be investigated (table 2).

Clinicians voiced concern that non-TWW patients with and without cancer were being disadvantaged by the priority given to TWW patients with cancer. This is an important issue, since the majority of patients diagnosed with CRC are referred and diagnosed via other routes,^27^ and the number of patients with other GI conditions outnumber those for CRC.^28^ While the effect of the rapid-referral route on survival remains inconclusive,^14^ ^27^ ^29^ patients with CRC waiting outside of the TWW referral system have been found to be waiting longer for treatment.^14^ There is also concern that diagnosis via the routine referral route may result in unnecessary anxiety for patients. Better symptom triage (see above) could improve the diagnostic process for patients presenting with atypical symptoms. Evidence of the effect of the priority given to patients with cancer on other GI patients is generally lacking and needs further investigation.

Clinicians noted that the emphasis given to the accomplishment of waiting time targets was on occasion detrimental to providing the best care. They raised the importance of input from front-line staff, including agreeing individual time frames for each patient. These views have also been reported elsewhere and are in contrast with the views of managers whose focus is on meeting national operational targets.^6^ Managers report concern about fines for target breaches, particularly in the face of constrained resources and increased demand for cancer care.^6^ The disagreement between the clinicians’ need for clinical autonomy and the administrators’ focus on meeting the standards was noted by previous studies to cause tension,^6^ and could have negative impacts on the morale and motivation of both sides. There is also concern on the costs and demand of consultant time for MDT meetings. New models of service delivery for cancer care, which strikes a balance between providing quality service while making efficiency savings are needed.^6^ This should include some flexibility in the implementation of the targets, within acceptable time frames. Local variations in cancer service delivery were reported, and should be evaluated.

Clinicians argued for targets to accommodate site-specific pathways, and to take into consideration complexities in diagnosis and treatment, suggesting this was crucial for CRC, where diagnosis involved invasive and time-consuming procedures. The 74% accomplishment rate (Q3 2014/2015) of the 62-day TWW target for lower GI cancers compared with the 99% for the 31-day target^10^ suggests a delay during the diagnostic and treatment initiation process. Our study highlights the time waiting for tests as a possible bottleneck in the pathway of patient with CRC. Delays in radiology, endoscopy and oncology, as well as waiting for clinic appointments and the start of treatment (either surgery or neoadjuvant therapy), have been cited in previous studies as stalling points.^14^ ^15^ ^28^ Delays were attributed to the lack of physical capacity (clinic space, diagnostic activity) to accommodate rising demand.^6^ Fragmentation of cancer services, possibly leading to gaps in patient care or duplication of processes, was also reported to impede prompt diagnosis and treatment.^6^ Increased capacity or better allocation of resources and improved coordination of care were proposed to counter this challenge. More in-depth studies and quantitative investigations are needed to assess this.

## Conclusion

After 10 years, a perception of challenges in the implementation of the CRC TWW referral guidelines and waiting time targets persist, producing unintended harms at the individual and organisational levels. Some recommendations and strategies suggested by the clinicians and reported in the literature could be, and are being, evaluated and implemented. The differing outcomes related to local variation in implementation should be urgently evaluated, and practices proven to be effective in some areas (such as audit and feedback, assignment of some investigations to primary care, increasing diagnostic capacity and having an online referral system) adopted more widely.
